# Predicting Blood Parasite Load and Influence of Expression of iNOS on the Effect Size of Clinical Laboratory Parameters in Acute *Trypanosoma cruzi* Infection With Different Inoculum Concentrations in C57BL/6 Mice

**DOI:** 10.3389/fimmu.2022.850037

**Published:** 2022-03-18

**Authors:** Wellington Francisco Rodrigues, Camila Botelho Miguel, Laís Corrêa Marques, Thiago Alvares da Costa, Melissa Carvalho Martins de Abreu, Carlo José Freire Oliveira, Javier Emilio Lazo-Chica

**Affiliations:** ^1^ Postgraduate Course in Health Sciences, Federal University of Triângulo Mineiro, Uberaba, Brazil; ^2^ Biosciences Unit, Centro Universitário de Mineiros, Mineiros, Brazil; ^3^ Postgraduate Course in Tropical Medicine and Infectology, Federal University of Triângulo Mineiro, Uberaba, Brazil; ^4^ Cell Biology Laboratory, Institute of Biological and Natural Sciences of the Federal University of Triângulo Mineiro, Uberaba, Brazil

**Keywords:** *Trypanosoma cruzi*, inoculum concentration, inducible nitric oxide synthetase, immunosuppression, statistical modeling

## Abstract

In Chagas disease, the initial responses of phagocyte-mediated innate immunity are strongly associated with the control of *Trypanosoma cruzi* and are mediated by various signaling pathways, including the inducible nitric oxide synthetase (iNOS) pathway. The clinical and laboratory manifestations of Chagas disease depend on the parasite–host relationship, i.e., the responsive capacity of the host immune system and the immunogenicity of the parasite. Here, we evaluated effect sizes in clinical and laboratory parameters mediated by acute infection with different concentrations of *T. cruzi* inoculum in mice immunosuppressed *via* iNOS pathway inactivation. Infection was induced in C57BL/6 wild-type and iNOS^-/-^ mice with the “Y” strain of *T. cruzi* at three inoculum concentrations (3 × 10^2^, 3 × 10^3^, and 3 × 10^4^). Parasitemia and mortality in both mouse strains were monitored. Immunohistochemistry was performed to quantify amastigotes in cardiac tissues and cardiac musculature cells. Biochemical parameters, such as blood urea nitrogen, sodium, albumin, and globulin concentrations, among others, were measured, and cytokine concentrations were also measured. Effect sizes were determined by the eta squared formula. Compared with that in wild-type animals, mice with an absence of iNOS expression demonstrated a greater parasite load, with earlier infection and a delayed parasitemia peak. Inoculum concentration was positively related to death in the immunosuppressed subgroup. Nineteen parameters (hematological, biochemical, cytokine-related, and histopathological) in the immunocompetent subgroup and four in the immunosuppressed subgroup were associated with parasitemia. Parasitemia, biochemical parameters, and hematological parameters were found to be predictors in the knockout group. The impact of effect sizes on the markers evaluated based on *T. cruzi* inoculum concentration was notably high in the immunocompetent group (Cohen’s *d* = 88.50%; *p* <.001). These findings contribute to the understanding of physiopathogenic mechanisms underlying *T. cruzi* infection and also indicate the influence of the concentration of *T. cruzi* during infection and the immunosuppression through the iNOS pathway in clinical laboratory heterogeneity reported in acute Chagas disease.

## Introduction

*Trypanosoma cruzi* is a protozoan with vast genetic variability and the ability to infect humans and cause Chagas disease (1, 2). It is estimated that around 6 to 7 million individuals worldwide, primarily in Latin America, are infected with *T. cruzi*, and chronic infection can cause cardiac alterations in 30% of patients or neurological or mixed alterations in 10% of patients (3).

While parasitic infection can occur *via* different mechanisms, its development depends on methods of invasion and intracellular division of the parasite. As part of the immune response, macrophages stand out among infected cells (4, 5). Parasites are internalized by macrophage phagosomes, wherein they may be eliminated or evade the cytosol to undergo replication, which subsequently disrupts host cells and can lead to infection of other cells or tissues (6, 7). Macrophages play a key role in inhibiting *T. cruzi* proliferation (8, 9).

The destruction of parasites within macrophages of the vertebrate host is mediated by certain known mechanisms, such as nitric oxide (NO) production (10, 11). Upon elevation in the levels of proinflammatory proteins, such as interferon gamma (IFN-γ), produced by natural killer lymphocytes in response to the initial immunostimulation mediated by innate immunity, the parasite can induce the trypanocidal activity of macrophages mediated by NO release in the phagosomes (12). Inducible NO synthase (iNOS) is responsible for NO synthesis. NO directly or indirectly modulates the effector functions aimed at eliminating the parasite, which involves trypanocidal effects mediated by an increase in the levels of toxic free radicals, such as peroxynitrite and superoxide, or aggravation of the proinflammatory response (10).

In contrast to the antiparasitic effects mediated by immune cell signaling processes, tissue damage is induced by the proinflammatory proteins produced in host cells (13). However, the lack of expression of proinflammatory cytokines in acute *T*. *cruzi* infection has been confirmed to serve as a limiting factor to survival as well as to infection suppression (14).

The intensity of the immune response to infection is variable and depends on factors such as the antigenic and/or phenotypic characteristics of the parasite (15, 16), the *T. cruzi* load during infection (17, 18), and the responsiveness of the host’s immune system (19). The inoculum concentration has previously been shown to interfere with the pathophysiology of the disease and modify the intensity of the outcomes observed in the heart (20), intestines (21), and kidneys (22) in experimental models. However, the intensity of effect sizes in clinical and laboratory parameters has not been reported, and the influence of immunosuppression mediated by the absence of iNOS pathway activation on systemic and cardiac effects is not well understood.

In general, the host–parasite relationship is directly associated with clinical and laboratory parameters observed at different stages of Chagas disease. These parameters are the result of inflammatory components generated after the invasion by *T. cruzi*, and limiting factors to the initial or acute phase characterization of the disease are discrepancies in or heterogeneity of clinical and laboratory parameters observed in *T. cruzi* infection (23). However, different biochemical markers or hematological parameters may consistently reflect the acute state of the disease in study models with controlled management provided that the inoculum concentration or parasite load, the type of parasite strain, and the characteristics of the host are established (22, 24, 25). This relationship is very useful because it makes it possible to assess the prognosis of the infection, where greater changes are associated with worse prognosis. The heterogeneity of the disease at first makes it difficult in clinical practice to relate the changes of laboratory markers with the characteristics of *T. cruzi* infection and the patient’s immune status. However, we believe that well-established experimental definitions of host–parasite relationships and laboratory markers may help in the development of future computational models or machine learning that will guide the diagnosis and prognosis of *T. cruzi* infection in humans, as already established for other diseases (26–28).

This study aimed to determine predictors of disease in a controlled model of acute *T. cruzi* infection by evaluating the relationship between acute infection-mediated effect sizes in clinical and laboratory parameters and different *T. cruzi* inoculum concentrations in mice with immunosuppression caused by iNOS pathway inactivation. The results helped to understand the aspects of the heterogeneity of the disease and its clinical laboratory relationships.

## Materials and Methods

### Experimental Design

An explanatory/analytical experimental study was performed in a mouse model of acute *T. cruzi* infection. All stages of the study were blinded. The animals in each group were allocated randomly using a table of random numbers. The animals were previously subjected to a one-week period of acclimatization in their new groups. Environmental enrichment was performed through the provision of sunflower seeds and changes in food distribution (height and disposition).

The animal groups underwent a new evaluation to ensure equality in weight and food intake between groups and to reduce confounding factors for outcomes post-infection. Research evaluations were performed by at least two evaluators with prior independent training, and data replicability was assessed using the kappa coefficient of agreement (kappa > 0.90). The survey results independently reflect data from duplicate experiments.

### Animals

Male C57BL/6 wild-type and iNOS^-/-^ mice (*n* = 80, 40 animals from each lineage), aged 10 weeks and weighing 20–30 g, were housed in temperature-controlled rooms (22–25°C) with *ad libitum* access to water and food (Nuvilab-CR1, NUVITAL, Nutrients Veterinary Products Ltda, Curitiba, PR, Brazil) in the animal facilities of the Laboratory of Cell Biology, Institute of Biological and Natural Sciences, Federal University of Triângulo Mineiro (UFTM), Uberaba, Minas Gerais, Brazil. The protocols for all experiments involving mice were evaluated and approved by the UFTM Institutional Animal Care and Use Committee (protocol number 293/2013). No mouse was included in more than one experimental group.

### Parasite Strain and Experimental Groups

The “Y” strain of *T. cruzi* was used in experimental studies. C57BL/6 mice (ten animals per group) were injected subcutaneously with blood-derived trypomastigotes (MHOM/BR/00Y; *T. cruzi* II) according to methods reported in a previous study (29, 30); the strain was kindly provided by the University of São Paulo (Brazil) and maintained at the Department of Cell Biology at UFTM. The mice used in this study were divided into the following groups: uninfected or infected with 3 × 10^2^ (low), 3 × 10^3^ (medium), or 3 × 10^4^ (high) trypomastigotes (20–22). Each group comprised ten animals.

### Parasitemia and Survival

Parasitemia was quantified in infected mice according to Brener’s technique (31). Briefly, were counted parasites present in 50 microscopic fields of a wet preparation containing 5 μL of blood collected from of the distal portion of the tail after cleaning and a small incision (around 3 mm) followed by dressing of the lesion. Microscopic blood parasite examinations were performed daily until day 12 of infection, and results were expressed as the number of parasites per milliliter. In other experiments, mice were infected with 3 × 10^2^, 3 × 10^3^, or 3 × 10^4^ trypomastigotes, and the animals were followed for 22 days to assess the outcome of death after infection. Each death was reported after finding cardiorespiratory arrest and absence of diaphragmatic contraction and arterial pulse.

### Sample Collection

We performed 24 h urine collection in metabolic cages (days 11 to 12 of infection). After 12 days of infection the animals were sacrificed (n = 10 animals for each subgroup were evaluated, total n = 80 animals). The animals were fasted for 4 h and heparinized afterwards with 40 units of Hemofol (5000 IU/mL). Unconsciousness and analgesia were induced with carbon dioxide, and all organs (including the heart) and blood (drawn through the ophthalmic plexus) were removed for evaluation. The euthanization procedures were performed in an environment different from the experimentation environment, with no contact among the animals during euthanasia. In addition, the euthanization process occurred simultaneously for at least one animal from each group, selected through randomization.

### Histological and Immunohistochemical Analyses

The mouse hearts were washed with 0.9% saline solution at 5°C, and a cross-sectional dissection was performed along the long axis of the ventricle, which produced a 2 mm-thick slice corresponding to one-third of the tissue. The slices were immediately inserted in a solution containing a protease inhibitor and frozen at -80°C for cytokine evaluation. The remaining two-thirds of the cardiac tissue was placed in methacarn for 24 h and then stored in 70% alcohol. Subsequently, the heart tissue was subjected to dehydration with an ethyl alcohol series and diaphanization in xylene and embedded in paraffin for microtomy. Sections with a thickness of 6 µm were obtained in a rotating microtome Leica RM2245 (Leica Microsystems, Wetzlar, Germany) and mounted on slides previously treated with a silane adhesive. The cuts were serialized at 60-µm intervals on the same slide, and 10 slides were obtained with four cuts in each. A part of the tissue was used for staining with hematoxylin (32), and the remaining tissue was used for immunohistochemistry.

After endogenous peroxidase and nonspecific binding blockade and antigen recovery, the sections were treated for 2 h with rabbit anti-*T. cruzi* antibody (dilution 1:250) at 25°C. Later, the slides were washed with PBS, treated with protein A conjugated with peroxidase (1:100), and developed with DAB-diaminobenzidine in Tris-HCl buffer (pH 7.4). The sections were counterstained with hematoxylin, and the slides were mounted with Entellan for analysis under an ordinary light microscope (33).

For morphometry, the inflammatory infiltrates and *T. cruzi* nests were quantified using a color digital video camera (Evolution MP 5.0, Media Cybernetics, Rockville, MD, USA) coupled to a light microscope (Eclipse 50i, Nikon, Kawasaki, Japan) that relayed the images to a computer. Images were captured using the ImagePro Plus program (Media Cybernetics) and analyzed using ImageJ software (http://rsb.info.nih.gov/ij/). The images (2560 × 1920 pixels) were calibrated using a blade (Leica) with a ruler with graduations of 2 mm divided into units of 10 µm for a 10× objective for immunohistochemistry and 20× objective for cell quantitation.

For the quantification of cardiac tissue cells and inflammatory infiltrate, 10 images selected at random were used and distributed equally in the right and left ventricular region of each animal in duplicate. Each image had dimensions of 724.45 µm × 543.34 µm and an area of 393,625.63 µm^2^. In total, an area of 3,936,256.3 µm^2^ was analyzed, corresponding to 3.93 mm^2^ per duplicate.

To determine the number of cells, the semi-automatic mode of ImageJ was used after the nuclei of cardiac tissue cells were identified. The cells in the uninfected group were used to standardize the mean number of cells in the tissue, following which the mice infected with different inoculum concentrations were evaluated. The result was expressed in terms of the ratio of the number of cells divided by the area in mm^2^.

To determine the number of *T. cruzi* nests, an average of 218 fields per animal were tested, corresponding to a total area of 10.67 mm^2^. Each area of a field corresponded to 48,858.16 µm^2^. The images were distributed in equal numbers in the right and left ventricular regions of each animal. In the end, the ratio of the area occupied by *T. cruzi* nests to the total area analyzed was determined, and the results were expressed in cm^2^.

### Blood Cell and Reticulocyte Count

After collection, 100 μL of whole blood was aliquoted into a tube containing 5 μL of 10% EDTA and analyzed using a hemocytometer (ABX MICROS 60, Horiba ABX Diagnostics, Montpellier, France). This device determined the following hematological parameters: red blood cell count, hematocrit, mean corpuscular volume, hemoglobin level, mean corpuscular hemoglobin, erythrocyte volume distribution amplitude, and total leukocyte count. The result corresponded to the average of two readings from the same sample. Next, blood smear slides stained with Panótico (NewProv, Pinhais, Brazil) were prepared for platelet counting and the leukocyte differential test. Subsequently, 15 μL of whole blood was incubated with 15 μL of brilliant cresyl blue dye (Laborclin, Campo Novo do Parecis, Brazil) at 37°C for 20 min to prepare the slides for reticulocyte counting.

### Evaluation of Biochemical Parameters

Blood and urine samples were centrifuged at 1831 × *g* at 4°C for 10 min to obtain the plasma and supernatant. The plasma concentrations of blood urea nitrogen (BUN), sodium, potassium, chlorine, glutamic-oxaloacetic transaminase (GOT), glutamic-pyruvic transaminase (SGPT), alkaline phosphatase (ALP), creatine phosphokinase (CPK), creatine kinase myocardial band, total protein, albumin, and globulin and the albumin-to-globulin ratio (A/G ratio) were determined. The urinary concentrations of sodium, potassium, chlorine, urea, and creatinine were measured. In addition, the glomerular filtration rate was estimated by determining creatinine clearance in mL/min × 0.006179 (34). Measurements were performed *via* spectrophotometry using an automated device (COBAS INTEGRA 400, Roche Diagnostics Corp., Indianapolis, IN, USA).

### Quality Control

In all stages of the study (pre-analytical, analytical, and post-analytical), internal quality control processes were implemented. The objectives, procedures, standards, criteria for tolerance limits, corrective actions, and registration of activities were reported, and the use of controls for evaluating the imprecision of analyses was stated and monitored. Control charts, such as the Levey-Jennings chart and multiple Westgard Rules, were also used (34, 35).

### Cytokine Measurement

Cytokines were measured using an enzyme-linked immunosorbent assay according to the manufacturer’s instructions (OptEIATM Kit, Pharmingen, San Diego, CA, USA). High-affinity polystyrene plates (Corning Costar Europe, Badhoevedorp, The Netherlands) were sensitized with a specific capture antibody for each cytokine (50 mL/well) diluted in 0.1 M carbonate–bicarbonate buffer (pH 9.6) and then incubated for 24 h at 4°C. Next, the plates were washed with PBS-T and incubated with 1× PBS along with 10% inactivated fetal bovine serum (blocking solution) (Sigma-Aldrich, St. Louis, MO, USA) for 1 h at 25°C. Known concentrations of recombinant cytokines (for the standard curve) and the sample to be analyzed were added to 96-well ELISA plates in duplicate and incubated at 25°C for 2 h. Subsequently, the plates were washed with PBS-T. Then, secondary biotinylated antibodies (detection antibody) for each cytokine, pre-incubated for 15 min with peroxidase-conjugated avidin, were diluted; this solution was added to the plates and incubated at 25°C for 1 h. After a washing step, tetramethylbenzidine (Pierce Biotechnology, Waltham, MA, USA) as a developer and H_2_O_2_ as substrate were added. The reactions were blocked after 20 min using 2 M sulfuric acid, and readings were measured at 450 nm by a microplate reader (Power Wave X, BioTek Instruments, Inc., Winooski, VT, USA). The concentrations of IL-12 p40, IL-10, IFN-γ, TNF-α, and IL-17 were measured with reference to the standard curve generated from serial dilutions of the recombinant cytokines. Cytokine concentration was expressed in pg/mL/g.

### Statistical Analysis

G*Power version 3.1.7 (Uiversität Kiel, Kiel, Germany) was used for sampling estimates and power of inferences. Data were tabulated in the Microsoft^®^ Excel program and analyzed using IBM SPSS Statistics 21 (IBM Corp., Armonk, NY, USA) and jamovi 1.6.15 (36, 37). Survival differences among groups were verified using the log-rank test. Parasitemia and the effects of different concentrations of *T. cruzi* inocula were evaluated with respect to distribution using the Shapiro–Wilk test and homoscedasticity using Levene’s test. Welch correction was used for cases of unequal variances. A one-way analysis of variance was used with Tukey’s post-test for equal variances or with the Games–Howell test for unequal variances. The effect size was determined using the eta squared (η^2^) formula.

A multinomial logistic regression model was used to predict and estimate the effects [odds ratios (OR) and confidence intervals (CIs)] of different inoculum concentrations on laboratory parameters and their association with the parasite load. The parasite load was subdivided into four potential categories: undetected (absence of parasites), light (50,000–200,000 parasites/mL), moderate (201,000–500,000 parasites/mL), and high (above 501,000 parasites/mL). Bionomial logistic regression modeling was also performed for the outcome of death in the immunosuppressed subgroup. The parameters selected for modeling fulfilled three criteria: the effects among different inoculum concentrations were statistically significant (*p* <.05), the power of inferences was >70%, and the parameter exhibited multicollinearity (tolerance of 80%). The Akaike information criterion (AIC), Schwarz Bayesian criterion (BIC), and R² of McKelvey were used to assess the complexity and adherence of the models.

Lastly, the relationship between the effect sizes of the two mouse strains was obtained for each parameter analyzed in the study. To compare possible differences in the distributions of effect sizes for each parameter, the distributions of delta variables (paired samples *t*-test) were evaluated, and the effect size was determined using Cohen’s *d*. A significance level of 5% was considered in all analyses (38, 39). The experimental design, database, methods and data analysis were confirmed by a statistician.

## Results

### Effects of Different *T. cruzi* Inocula on Parasitemia and Survival of Wild-Type and iNOS^-/-^ C57BL/6 Mice

**Figure 1:**
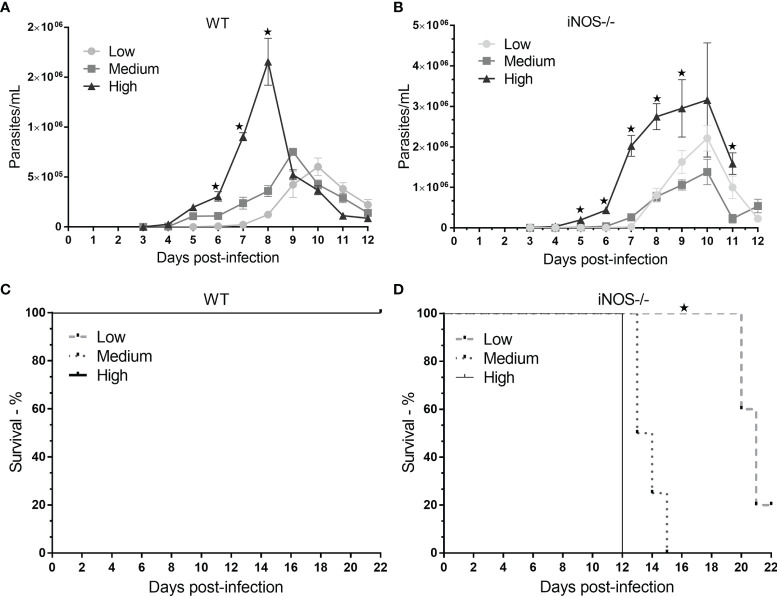
Evaluation of parasitemia and survival in wild-type (WT) and knockout (iNOS^-/-^) mice infected with different *Trypanosoma cruzi* inoculum concentrations. C57Bl/6 WT and iNOS^-/-^ mice were infected with low (3 × 10^2^), medium (3 × 10^3^), or high (3 × 10^4^) trypomastigote inoculum concentrations. In **(A, B)**, parasitemia was evaluated daily for the different profiles and research groups for 12 days. The number of parasites per milliliter was expressed in terms of mean and standard deviation. **(C, D)** shows the survival of the subgroups; the animals were followed until day 22 (censorship point), with the same experimental conditions for each phenotypic profile and inoculum. Survival is represented in percentages. The * represents significant differences (*p* <.05) (ANOVA followed by Tukey’s or Games–Howell test for parasitemia and log-rank test for survival).

Parasitemia was evaluated daily until day 12 of infection. Throughout the evaluation period, the mean distribution of the number of parasites/mL was greater for the highest inoculum concentration than for the lower inoculum concentrations for both mouse strains. On the third day after infection, the high groups in both mouse strains tested positive, confirming the presence of *T. cruzi*. The number of iNOS^-/-^ animals that tested positive was greater than 3× the number of wild-type animals that tested positive. The differences in parasitemia between the two mouse strains followed during the experimentation period. Statistically significant differences between the iNOS^-/-^ profile in the high group and that in the low group were reported on day 5, whereas for the wild-type profile, the same differences were observed on day 6 after infection. The permanence of the parasite load in the iNOS^-/-^ group culminated in the peak of parasitemia on day 10 after infection, whereas in the wild-type group, the peak was inoculum-dependent on days 8 (high), 9 (medium), and 10 (low) (**Figures 1A, B**).

After the parasitemia of each strain was determined, the survival of infected animals was reported until day 22 in a different experiment (**Figures 1C, D**). The survival pattern was similar between the different experiments. There were no deaths in the wild-type groups, whereas, in the knockout group, 100% of the animals died in the high and medium subgroups after 12 and 15 days of infection, respectively, and 80% of the deaths observed in the low subgroup had occurred by day 22 after infection (*p* <.05).

### Influence of *T. cruzi* Inoculum Concentration on the Effects of Differences in Clinical Laboratory Markers, Inflammatory, and Histopathological Parameters

Different clinical laboratory markers, inflammatory, and histopathological parameters were evaluated in experimental models of acute Chagas disease to determine the statistical effect (η^2^) of different parasite loads on infection in immunocompetent and immunosuppressed mice (**Table 1**). The greatest changes were observed in the subgroups that received a high load of of *T. cruzi*. In the wild-type C57BL/6 mice lineage, infection was related to a significant increase (*p* <.05) in BUN levels, plasma GOT, plasma globulin, reticulocytes, and monocytes in blood and inflammatory cytokine levels (TNF-α, IL -10, IFN-γ, IL-12p40, and IL-17) and the number of cells and amastigote nests in cardiac tissues. These alterations were inoculum-dependent. Reduction in different parameters dependent on the *T. cruzi* inoculums, such as plasma potassium, plasma SGPT, plasma ALP, plasma total protein, plasma albumin, A/G ratio, urinary sodium, urinary potassium, urinary urea, erythrocytes, hemoglobin, hematocrit, total leukocyte count, and lymphocyte count (*p* <.05), was also observed.

**Table 1:** Effects of different *Trypanosoma cruzi* inoculum concentrations (low, medium, and high) of the “Y” strain on clinical laboratory markers, inflammatory, and histopathological parameters in wild-type C57BL/6 and knockout (iNOS^-/-^) mice in the acute phase of infection (after 12 days of infection).

**Wild-type C57BL/6 mice**								
**Parameter**	**Uninfected **	***T. cruzi* inoculum**			**F**	***p*-value**	**η^2^ - %**	**Power - %**
	**Control – mean ± SD **	**Low – mean ± SD**	**Medium – mean ± SD**	**High – mean ± SD**				
BUN* - mg/dL	17.81 ± 0.90^a^	18.41 ± 4.04^a^	21.98 ± 0.09^ab^	24.77 ± 1.74^b^	25.97	0.002	67.70	94.03
Plasma sodium - mmol/L	165.25 ± 11.81^a^	151.25 ± 1.26^a^	150.50 ± 1.29^a^	150.00 ± 0.82^a^	2.50	0.153	60.00	86.81
Plasma potassium - mmol/L	7.51 ± 0.51^ac^	6.21 ± 0.17^bc^	7.28 ± 0.96^abc^	6.71 ± 0.46^c^	7.96	0.018	49.20	69.41
Plasma chlorine - mmol/L	122.82 ± 9.29^a^	112.20 ± 0.59^a^	112.81 ± 2.37^a^	115.46 ± 1.87^a^	4.30	0.068	49.80	70.58
Plasma GOT* - U/L	245.59 ± 69.99^a^	573.44 ± 70.57^b^	471.65 ± 29.72^b^	556.89 ± 121.06^b^	13.89	0.004	78.20	98.46
Plasma SGPT - U/L	59.43 ± 18.30^ab^	89.71 ± 15.16^a^	81.24 ± 49.17^ab^	38.58 ± 9.52^b^	9.38	0.010	40.70	51.42
Plasma ALP* - U/L	127.93 ± 37.76^a^	105.58 ± 15.98^a^	81.81 ± 22.83^ab^	52.62 ± 2.50^b^	16.92	0.004	65.40	92.30
Plasma CPK - U/L	793.86 ± 406.49^a^	5455.75 ± 3205.45^a^	4127.50 ± 4519.08^a^	2696.75 ± 1661.53^a^	3.95	0.082	32.20	33.55
Plasma CKMB - U/L	505.00 ± 33.16^a^	807.00 ± 205.17^a^	799.50 ± 339.15^a^	658.50 ± 130.27^a^	4.22	0.073	31.70	32.60
Total plasma protein* - g/dL	5.33 ± 0.150^a^	5.16 ± 0.12^a^	5.01 ± 0.30^ab^	4.68 ± 0.20^b^	7.50	0.016	63.60	90.70
Plasma albumin* - g/dL	4.21 ± 0.99^a^	2.99 ± 0.07^b^	2.74 ± 0.26^b^	2.44 ± 0.05^b^	41.94	< .001	69.20	94.98
Plasma globulin* - g/dL	1.87 ± 0.04^a^	2.15 ± 0.20^b^	2.31 ± 0.05^b^	2.25 ± 0.14^b^	22.51	0.001	68.40	94.49
Plasma A/G ratio*	1.84 ± 0.10^a^	1.38 ± 0.03^b^	1.21 ± 0.14^bc^	1.09 ± 0.10^c^	31.63	< .001	91.20	99.82
Urinary sodium - mmol/L	119.60 ± 35.71^a^	83.60 ± 9.76^ab^	120.40 ± 21.96^a^	80.20 ± 20.37^b^	4.67	0.036	44.50	59.69
Urinary potassium* - mmol/L	734.94 ± 160.61^a^	430.34 ± 134.31^b^	423.86 ± 68.07^b^	325.72 ± 96.15^b^	6.90	0.012	67.20	93.68
Urinary chlorine - mmol/L	345.12 ± 70.90^a^	272.78 ± 67.38^a^	310.39 ± 35.21^a^	228.43 ± 70.18^a^	2.43	0.137	37.50	44.46
Urinary urea* - mg/dL	11487.06 ± 1575.97^a^	7426.72 ± 2090.38^b^	7759.28 ± 1501.89^b^	6526.02 ± 1935.84^b^	7.43	0.009	58.30	84.63
Urinary creatinine - mg/dL	37.10 ± 3.22^a^	35.76 ± 9.01^a^	39.11 ± 5.86^a^	33.46 ± 10.06^a^	0.33	0.807	8.50	6.57
Crcl - (mL/min)×0.006179	0.12 ± 0.01^a^	0.12 ± 0.01^a^	0.10 ± 0.03^a^	0.11 ± 0.02^a^	0.63	0.620	14.70	9.99
Erythrocytes* - mm^3^	8970000 ± 428311^a^	7780000 ± 178939^b^	7590000 ± 40000^b^	6390000 ± 41446^c^	678.92	< .001	95.00	99.91
Hemoglobin* - g/dL	13.28 ± 0.72^a^	11.68 ± 0.16^b^	11.20 ± 0.25^b^	9.92 ± 0.31^c^	46.55	< .001	91.00	99.81
Hematocrit* - %	45.38 ± 2.90^a^	37.20 ± 0.85^b^	35.75 ± 0.26^b^	30.20 ± 0.62^c^	120.82	< .001	93.90	99.89
MCV - μm^3^	50.80 ± 2.38^a^	47.60 ± 1.14^a^	47.05 ± 0.71^a^	47.20 ± 0.83^a^	3.38	0.071	59.00	85.56
MCH - pg	14.78 ± 0.62^a^	15.00 ± 0.21^a^	14.80 ± 0.25^a^	15.47 ± 0.52^a^	1.77	0.236	31.80	32.78
Reticulocytes* - mm^3^	309138 ± 106849^ab^	376240 ± 67939^b^	234055 ± 55029^a^	189335 ± 33707^a^	9.52	0.005	55.80	81.04
Total leukocytes* - mm^3^	7640 ± 1799^a^	4620 ± 1158^b^	6875 ± 936^a^	5934 ± 830^ab^	4.46	0.037	50.80	72.49
Lymphocytes* - mm^3^	6717.40 ± 1513^a^	3502.00 ± 982^b^	4938.75 ± 834^ab^	4635.60 ± 868^b^	4.84	0.029	58.60	85.03
Monocytes* - mm^3^	355.00 ± 401^a^	357.80 ± 146^a^	1258.50 ± 307^b^	792.93 ± 111^ab^	14.54	0.001	70.70	95.81
Neutrophils - mm^3^	567.60 ± 291^a^	760.20 ± 246^a^	677.75 ± 194^a^	514.00 ± 117^a^	1.59	0.263	18.90	13.66
TNF-α* - pg/mL/g	64.25 ± 34.91^a^	100.00 ± 21.60^a^	302.50 ± 84.60^b^	422.50 ± 55.60^c^	40.82	< .001	90.60	99.80
IL-10* - pg/mL/g	108.75 ± 65.36^a^	182.50 ± 100.78^a^	502.50 ± 214.53^b^	590.00 ± 104.24^b^	18.86	0.001	75.70	97.81
IFN-γ* - pg/mL/g	137.50 ± 109.65^a^	172.50 ± 95.35^a^	480.00 ± 132.91^b^	645.00 ± 174.45^b^	10.63	0.006	77.60	98.33
IL-12p40* - pg/mL/g	182.50 ± 99.79^a^	132.50 ± 85.00^a^	457.50 ± 105.31^b^	612.50 ± 85.39^b^	22.05	< .001	85.40	99.50
IL-17* - pg/mL/g	182.50 ± 92.51^ab^	137.50 ± 66.52^a^	343.75 ± 109.95^b^	595.00 ± 101.48^c^	17.22	0.002	82.90	99.25
Cell number* - mm^2^	2113.43 ± 71.63^a^	2223.38 ± 179.80^ab^	2400.52 ± 65.64^b^	2448.16 ± 205.15^b^	12.01	0.003	49.80	70.58
Number of nests - cm^2^	—————	33.63 ± 18.80^a^	168.17 ± 214.37^b^	225.98 ± 54.36^b^	4.70	0.015	46.80	64.56
**Knockout mice (iNOS^-/-^)**								
**Parameter**	**Uninfected **	***T. cruzi* inoculum**			**F**	***p*-value**	**η^2^ - %**	**Power - %**
	**Control mean ± SD **	**Low – mean ± SD**	**Medium – mean ± SD**	**High – mean ± SD**				
BUN* - mg/dL	17.69 ± 2.38^a^	21.21 ± 4.23^ab^	19.99 ± 2.82^ab^	22.15 ± 2.78^b^	3.509	0.043	22.4	17.66
Plasma sodium - mmol/L	148.57 ± 5.62^a^	149.00 ± 5.29^a^	146.50 ± 4.95^a^	149.00 ± 4.21^a^	0.536	0.664	5.1	5.55
Plasma potassium - mmol/L	6.27 ± 0.62^a^	7.10 ± 1.03^ab^	7.74 ± 0.91^b^	7.45 ± 1.07^ab^	5.543	0.008	26	22.72
Plasma chlorine - mmol/L	109.09 ± 4.77^a^	109.12 ± 4.31^a^	110.03 ± 2.80^a^	112.96 ± 5.17^a^	1.086	0.384	13.3	9.02
Plasma GOT* - U/L	383.14 ± 149.01^a^	595.729 ± 82.55^a^	653.54 ± 124.86^a^	1652.33 ± 742.07^b^	8.049	0.004	66.7	93.32
Plasma SGPT - U/L	93.587 ± 36.55^a^	160.154 ± 52.53^a^	135.702 ± 16.76^a^	131.78 ± 26.69^a^	2.796	0.089	32.5	34.14
Plasma ALP* - U/L	161.60 ± 18.04^a^	126.56 ± 17.42^a^	113.26 ± 19.53^a^	228.29 ± 88.06^b^	11.843	< .001	50.7	72.3
Plasma CPK - U/L	747.67 ± 363.37^a^	1587.00 ± 721.67^b^	652.571 ± 289.57^a^	902.44 ± 649.80^ab^	3.552	0.042	33.2	35.52
Plasma CKMB - U/L	623.75 ± 257.24^a^	574.86 ± 131.96^a^	484.60 ± 72.78^a^	540.80 ± 133.25^a^	0.903	0.481	10.7	7.54
Total plasma protein* - g/dL	5.74 ± 0.50^a^	6.33 ± 0.28^a^	5.87 ± 0.36^a^	6.91 ± 0.58^b^	8.382	0.002	55.8	81.04
Plasma albumin* - g/dL	3.71 ± 0.26^a^	3.52 ± 0.20^a^	2.70 ± 0.06^b^	2.60 ± 0.15^b^	61.961	< .001	89.1	99.74
Plasma globulin* - g/dL	2.02 ± 0.27^a^	2.79 ± 0.32^b^	3.06 ± 0.29^b^	4.26 ± 0.58^c^	32.503	< .001	82.7	99.23
Plasma A/G ratio*	1.84 ± 0.16^a^	1.20 ± 0.08^b^	0.93 ± 0.10^c^	0.62 ± 0.09^d^	105.422	< .001	94.9	99.91
Urinary sodium - mmol/L	118.00 ± 54.64^a^	165.40 ± 39.56^a^	146.70 ± 13.21^a^	130.50 ± 32.98^a^	1.92	0.171	20.5	15.38
Urinary potassium* - mmol/L	343.80 ± 109.06^a^	378.71 ± 76.78^a^	356.83 ± 75.24^a^	361.66 ± 70.83^a^	0.228	0.876	2.5	5.13
Urinary chlorine - mmol/L	216.50 ± 64.25^a^	252.94 ± 40.97^a^	272.52 ± 44.22^a^	271.49 ± 61.62^a^	1.526	0.245	16.3	11.25
**Knockout mice (iNOS^-/-^)**								
**Parameter**	**Uninfected **	***T. cruzi* inoculum**			**F**	***p*-value**	**η^2^ - %**	**Power - %**
	**Control mean ± SD **	**Low – mean ± SD**	**Medium – mean ± SD**	**High – mean ± SD**				
Urinary urea* - mg/dL	6574.46 ± 3492.36^a^	9355.19 ± 1995.23^a^	8618.22 ± 1601.52^a^	8700.05 ± 836.11^a^	1.133	0.367	19.7	14.5
Urinary creatinine - mg/dL	31.11 ± 7.12^a^	46.47 ± 14.91^b^	44.93 ± 7.60^ab^	53.78 ± 9.92^b^	9.244	< .001	36.1	41.48
Crcl - (mL/min)×0.006179	0.11 ± 0.03^a^	0.12 ± 0.06^a^	0.14 ± 0.04^a^	0.16 ± 0.14^a^	0.771	0.527	5.4	5.62
Erythrocytes* - mm^3^	8320000 ± 1660000^a^	8270000 ± 1180000^a^	7050000 ± 1350000^a^	7330000 ± 492525^a^	1.774	0.207	18.7	13.46
Hemoglobin* - g/dL	13.73 ± 1.66^a^	12.58 ± 1.31^ab^	10.61 ± 1.90^b^	11.01 ± 0.78^b^	6.782	0.006	45.1	60.98
Hematocrit* - %	39.46 ± 7.52^a^	39.02 ± 3.84^ab^	33.53 ± 3.70^ab^	32.80 ± 1.86^b^	4.727	0.022	31.4	32.03
MCV - μm3	46.75 ± 0.89^ab^	47.60 ± 2.70^b^	45.30 ± 1.49^a^	44.71 ± 0.76^a^	7.843	0.004	36.6	42.54
MCH - pg	15.18 ± 0.31^a^	15.34 ± 0.72^a^	15.11 ± 0.72^a^	15.00 ± 0.40^a^	0.436	0.731	4.2	5.37
Reticulocytes* - mm^3^	173412 ± 27982^a^	415620 ± 167909^b^	347732 ± 62893^ab^	298283 ± 147594^ab^	11.842	0.003	41.7	53.61
Total leukocytes* - mm^3^	6413 ± 2315^ac^	11240 ± 2873^abc^	10870 ± 2918^b^	7867 ± 4446^bc^	5.316	0.013	29.1	27.83
Lymphocytes* - mm^3^	5713 ± 1954^a^	5884 ± 1181^a^	7509 ± 1678^a^	6082 ± 3367^a^	1.947	0.167	11	7.68
Monocytes* - mm^3^	271 ± 213^a^	1696 ± 520^bc^	1589 ± 972^bc^	1014 ± 522^ac^	16.925	< .001	44.6	59.91
Neutrophils - mm^3^	429 ± 268^a^	3660 ± 1374^b^	1771 ± 1663^a^	771 ± 810^a^	9.91	0.002	49.8	70.58
TNF-α* - pg/mL/g	516.68 ± 429.85^a^	349.79 ± 270.79^a^	385.69 ± 206.32^a^	156.66 ± 65.55^a^	3.39	0.068	18	12.77
IL-10* - pg/mL/g	328.82 ± 184.15^a^	202.71 ± 157.73^a^	232.13 ± 93.34^a^	198.03 ± 74.51^a^	0.701	0.572	12.8	8.7
IFN-γ* - pg/mL/g	12.45 ± 3.73^a^	12.07 ± 4.69^a^	14.21 ± 4.31^a^	10.72 ± 3.21^a^	0.638	0.608	8.3	6.49
IL-12p40* - pg/mL/g	134.66 ± 95.99^a^	119.27 ± 65.39^a^	125.40 ± 61.64^a^	67.47 ± 19.87^a^	2.775	0.103	13.6	9.22
IL-17* - pg/mL/g	233.86 ± 132.75^a^	163.59 ± 142.80^a^	152.36 ± 110.86^a^	70.00 ± 37.67^a^	3.225	0.074	19	13.76
Cell number* - mm^2^	2548 ± 140^a^	2479 ± 118^a^	2591 ± 317^a^	2692 ± 199^a^	2.525	0.091	13	8.83
Number of nests - cm^2^	—————	126.13 ± 61.29^ab^	252.26 ± 107.19^b^	475.08 ± 200.86^c^	20.9	< .001	76.8	98.12

BUN, blood urea nitrogen; GOT, glutamate oxaloacetate transaminase; SGPT, glutamate pyruvate transaminase; ALP, alkaline phosphatase; CPK, creatine phosphokinase; CKMB, creatine kinase myocardial band; A/G ratio, albumin to globulin ratio; Crcl, creatinine clearance; MCV, mean corpuscular volume; MCH, mean corpuscular hemoglobin; TNF, tumor necrosis factor; IL, interleukin; IFN, interferon. * = parameters with p <.05 and power above 70% in comparisons. SD, standard deviation. F, F-test values. η2, effect size estimation. Power, estimation of the power of inference. The letters a, b, c, and d indicate significant differences between the groups (ANOVA with Tukey’s or Games–Howell tests).

In the population of immunosuppressed animals (iNOS^-/-^), similar to that in the population of immunocompetent animals, inoculum-dependent increases in the levels of BUN, plasma GOT, and plasma globulin and the numbers of reticulocytes, monocytes, and amastigote nests were observed, alongside decreases in plasma albumin, A/G ratio, hemoglobin, and hematocrit (*p* <.05). However, unlike the wild-type animal subgroup, the knockout animal subgroup showed significant inoculum concentration-dependent increases in the levels of plasma potassium, ALP, CPK, and total protein; urinary creatinine; total leukocytes; and neutrophilic cell count (*p* <.05) with the onset of infection. The power for each inference was determined, as was the effect size for the observed differences (**Table 1**).

### Association of Clinical Laboratory Markers, Inflammatory, and Histopathological Parameters With the *T. cruzi* Load in Acute Infection in the Blood of Wild-Type C57BL/6 and Knockout (iNOS^-/-^) Mice Infected With Different Inoculum Concentrations

After evaluating and determining the effects of different *T. cruzi* inoculum concentrations on clinical and laboratory parameters, the variables that showed significant differences between the subgroups with an estimated power ≥70% were selected to compose a predictive model of the rates of *T. cruzi* infection in the blood; the reticulocyte parameter was excluded based on the collinearity effect presented in the distributions (**Table 2**).

**Table 2:** Estimation of parameters in a multinomial logistic regression model for the classification of *Trypanosoma cruzi* (strain “Y”) load in the blood (undetected, light, moderate, and high) in wild-type C57BL/6 and knockout (iNOS^-/-^) mice.

Wild-type C57BL/6 mice					
Parasitemia_classification	Predictor (Parameter)	Odds ratio	95% Confidence Interval		*p*-value
Moderate - Light			Lower	Upper	
	BUN - mg/dL	1.0966	1.0964	1.0969	< .001
	Plasma GOT - U/L	0.827	0.8191	0.835	< .001
	Plasma ALP - U/L	0.4308	0.4304	0.4312	< .001
	Total plasma protein - g/dL	0.8265	0.8265	0.8265	< .001
	Plasma albumin - g/dL	0.7446	0.7446	0.7446	< .001
	Plasma globulin - g/dL	0.8358	0.8358	0.8358	< .001
	Plasma A/G ratio	1.1162	1.1161	1.1162	< .001
	Urinary potassium - mmol/L	1.0413	1.0354	1.0473	< .001
	Urinary urea - mg/dL	0.9872	0.9075	1.074	0.765
	Erythrocytes - mm^3^	1	0.9999	1.0002	0.485
	Hemoglobin - g/dL	0.6049	0.6049	0.605	< .001
	Hematocrit - %	0.239	0.239	0.239	< .001
	Total leukocytes - mm^3^	1.0738	1.0274	1.1222	0.002
	Lymphocytes - mm^3^	0.9277	0.8874	0.9697	< .001
	Monocytes - mm^3^	0.9135	0.9023	0.9249	< .001
	TNF-α - pg/mL/g	1.0109	1.0036	1.0182	0.003
	IL-10 - pg/mL/g	0.905	0.8965	0.9136	< .001
	IFN-γ - pg/mL/g	1.0448	1.0355	1.0542	< .001
	IL-12p40 - pg/mL/g	1.0504	1.0412	1.0596	< .001
	IL-17 - pg/mL/g	1.1519	1.1422	1.1617	< .001
	Cell number - mm^2^	0.9608	0.9472	0.9745	< .001
**Not Detected - Light**					
	BUN - mg/dL	0.0325	0.0325	0.0325	< .001
	Plasma GOT - U/L	0.918	0.9091	0.927	< .001
	Plasma ALP - U/L	0.8327	0.8319	0.8334	< .001
	Total plasma protein - g/dL	0.997	0.997	0.997	< .001
	Plasma albumin - g/dL	0.415	0.415	0.415	< .001
	Plasma globulin - g/dL	1.0981	1.0981	1.0982	< .001
	Plasma A/G ratio	0.9879	0.9879	0.9879	< .001
	Urinary potassium - mmol/L	1.0883	1.0819	1.0947	< .001
	Urinary urea - mg/dL	0.9902	0.9082	1.0795	0.823
	Erythrocytes - mm^3^	1	0.9999	1.0002	0.514
	Hemoglobin - g/dL	0.3925	0.3925	0.3925	< .001
	Hematocrit - %	0.1281	0.1281	0.1281	< .001
	Total leukocytes - mm^3^	1.0211	0.9754	1.0688	0.372
	Lymphocytes - mm^3^	0.9746	0.9314	1.0198	0.266
	Monocytes - mm^3^	0.9652	0.9532	0.9774	< .001
	TNF-α - pg/mL/g	0.9633	0.9562	0.9706	< .001
	IL-10 - pg/mL/g	0.9126	0.9038	0.9214	< .001
	IFN-γ - pg/mL/g	1.0499	1.0407	1.0592	< .001
	IL-12p40 - pg/mL/g	1.0892	1.0796	1.0989	< .001
	IL-17 - pg/mL/g	1.1993	1.189	1.2097	< .001
	Cell number - mm^2^	0.9453	0.9319	0.959	< .001
**Knockout mice (iNOS^-/-^)**					
**Parasitemia_classification**	**Predictor (Parameter)**	**Odds ratio**	**95% Confidence Interval**		***p*-value**
**High – Not Detected**	** **		**Lower**	**Upper**	
	Plasma GOT - U/L	1.020	1.002	1.036	0.030
	Plasma ALP - U/L	0.965	0.901	1.035	0.319
	Total plasma protein - g/dL	37.530	2.190	64.350	0.012
	Plasma albumin - g/dL	0.012	0.008	0.650	0.031
	Plasma globulin - g/dL	19.760	2.228	167.330	0.032
	Plasma A/G ratio	0.030	0.005	14.080	0.699
	Hemoglobin - g/dL	0.355	0.160	0.787	0.011
	Neutrophils - mm^3^	1.002	0.998	1.004	0.076
**Moderate-Not Detected**					
	Plasma GOT - U/L	1.011	0.995	1.027	0.187
	Plasma ALP - U/L	0.889	0.833	0.950	< .001
	Total plasma protein - g/dL	4.860	0.385	61.280	0.222
	Plasma albumin - g/dL	0.084	0.021	0.875	0.047
	Plasma globulin - g/dL	42.150	0.575	309.450	0.076
	Plasma A/G ratio	0.070	0.010	9.410	0.699
	Hemoglobin - g/dL	0.274	0.109	0.692	0.006
	Neutrophils - mm^3^	1.002	0.995	1.003	0.149

BUN, blood urea nitrogen; GOT, glutamate oxaloacetate transaminase; ALP, alkaline phosphatase; A/G ratio, albumin to globulin ratio; TNF, tumor necrosis factor; IL, interleukin; IFN, interferon; Inf, infinite; Not detected = absence of parasites (by the method applied). Light = 50,000–200,000 parasites/mL. Moderate = 201,000–500,000 parasites/mL. High = above 501,000 parasites/mL. Odds ratio and 95% confidence interval = They indicate the estimate for the predictor effect size in a confidence interval associated with the blood parasite load outcome.

Given the large number of parameters, the model was considerably complex. For the wild-type C57BL/6 group, AIC = 64, BIC = 111, and R² of McKelvey = 1, the “high” categorie was not found. The model could be fitted better using only parameters directly related to immunity, such as leukocyte count and cytokine levels (AIC = 40; BIC = 69.30). In the knockout mice group, the model showed AIC = 36, BIC = 56.40, and R² of McKelvey = 1, the “light” category was not found. In the knockout group, the model was affected by numerical variations within each explanatory variable. Heterogeneity within the explanatory variables caused amplitude expansion of confidence intervals. The variability was a reflection of the immunological imbalance expected for the biological model of infection.

In the immunocompetent group, animals with mild and moderate parasitemia could be identified. Increases in some parameters were related to an increased likelihood of enhanced parasitemia for the moderate classification (201 to 500 thousand parasites/mL). The moderate classification of parasitemia was associated with increased BUN levels, A/G ratio, urinary potassium, total leukocyte content, and levels of the cytokines TNF-α, IFN-γ, IL-12p40, and IL-17 (**Table 2**). Furthermore, decreases in some parameters, such as plasma GOT, plasma ALP, total plasma protein, plasma albumin, plasma globulin, hemoglobin, hematocrit, lymphocyte count, monocyte count, IL-10, and cardiac tissue cell number, were linked to greater chances of more severe parasitemia under the moderate classification (*p* <.001) (**Table 2**).

Decreases in BUN levels, plasma GOT, plasma ALP, plasma total protein, plasma albumin, the A/G ratio, hemoglobin, hematocrit, monocytes, TNF-α levels, IL-10 levels, and cardiac tissue cell number and increases in plasma globulin, urinary potassium, IFN-γ levels, IL-12p40 levels, and IL-17 levels were used to distinguish animals without parasite detection from animals presenting with mild parasitemia (*p* <.001) (**Table 2**).

In the immunosuppressed group, the increase in plasma GOT, total plasma protein and plasma globulin levels was related to an increase in parasitemia to levels >501,000 per mL (high classification) (*p* <.05). Furthermore, decreased albumin and hemoglobin were associated with an high parasite load in the blood (*p* <.05). The plasma ALP, albumin and hemoglobin showed good sensitivity to the increase in parasitemia since its reduction was related to moderated blood parasite loads (p <.05) (**Table 2**).

### Association of Laboratory Parameters With the Outcome of Death in Mice With iNOS-Mediated Immunosuppression

The association of specific laboratory parameters with the outcome of death in the knockout animal subgroup could be estimated after the relationship of *T. cruzi* infection with different inoculum concentrations and the effect sizes for each of these variables were determined. A power of inferences ≥70% was used. Ten parameters were used, and although not all variables were directly associated with death, the model showed good explanatory adherence (AIC = 22, BIC = 34.5, and R² of McKelvey = 1).

The *T. cruzi* load in the blood (the mean number of parasites during infection) was a determining factor for death, as an increase in the number of parasites by 1 increased the chances of death by 1%. Elevations in plasma GOT levels and the plasma globulin ratio were also positively associated with death. Conversely, a protective effect was associated with increases in the plasma albumin concentration, A/G ratio, and hemoglobin levels, whereas decreases in these parameters were associated with death (*p* <.05) (**Table 3**).

**Table 3:** Binomial logistic regression model to estimate parameters associated with the outcome of death in knockout mice (iNOS^-/-^) infected with different inoculum concentrations of *Trypanosoma cruzi* (“Y” strain) in the acute phase (after 12 days of infection).

Knockout mice (iNOS^-/-^) - deaths				
Predictor (Parameter)	Odds ratio	95% Confidence Interval		*p*-value
		Lower	Upper	
Parasites/mL	1.010	1.000	1.013	0.008
Plasma GOT - U/L	1.010	1.000	1.020	0.034
Plasma ALP - U/L	1.008	0.995	1.020	0.209
Total plasma protein - g/dL	4.020	0.926	17.440	0.063
Plasma albumin - g/dL	0.093	0.013	0.681	0.019
Plasma globulin - g/dL	8.840	1.420	54.942	0.019
Plasma A/G ratio	0.013	4.03E-04	0.388	0.012
Hemoglobin - g/dL	0.527	0.300	0.926	0.026
Neutrophils - mm^3^	1.001	1.000	1.000	0.100
Cell number - mm^2^	1.001	0.998	1.000	0.471

GOT, glutamate oxaloacetate transaminase; ALP, alkaline phosphatase; A/G ratio, albumin to globulin ratio. Odds ratio and 95% Confidence Interval = They indicate the estimate for the predictor effect size in a confidence interval, associated with the death outcome.

### Effect of iNOS Immunosuppression on Clinical Laboratory Markers, Inflammatory, and Histopathological Parameters

After the effect sizes to each parameter evaluated under acute *T. cruzi* infection in each subgroup (wild-type and knockout) were determined, the impact of immunosuppression on each effect size could be assessed (**Table 4**).

**Table 4:** Determination of differences in the effects of *Trypanosoma cruzi* inoculum concentrations (low, medium, and high) of “Y” strain on clinical laboratory markers, inflammatory, and histopathological parameters between wild-type and knockout (iNOS^-/-^) mice with acute infection.

Parameter	Wild-type mice	Knockout mice	Ratio (Wild-type/Knockout)
	η2 - %	η2 - %	
BUN - mg/dL	67.70	22.4	3.02
Plasma sodium - mmol/L	60.00	5.1	11.76
Plasma potassium - mmol/L	49.20	26	1.89
Plasma chlorine - mmol/L	49.80	13.3	3.74
Plasma GOT - U/L	78.20	66.7	1.17
Plasma SGPT - U/L	40.70	32.5	1.25
Plasma ALP - U/L	65.40	50.7	1.29
Plasma CPK - U/L	32.20	33.2	0.97
Plasma CKMB - U/L	31.70	10.7	2.96
Total plasma protein - g/dL	63.60	55.8	1.14
Plasma albumin - g/dL	69.20	89.1	0.78
Plasma globulin - g/dL	68.40	82.7	0.83
Plasma A/G ratio	91.20	94.9	0.96
Urinary sodium - mmol/L	44.50	20.5	2.17
Urinary potassium - mmol/L	67.20	2.5	26.88
Urinary chlorine - mmol/L	37.50	16.3	2.30
Urinary urea - mg/dL	58.30	19.7	2.96
Urinary creatinine - mg/dL	8.50	36.1	0.24
Crcl - (mL/min) × 0.006179	14.70	5.4	2.72
Erythrocytes - mm^3^	95.00	18.7	5.08
Hemoglobin - g/dL	91.00	45.1	2.02
Hematocrit - %	93.90	31.4	2.99
MCV - μm^3^	59.00	36.6	1.61
MCH - pg	31.80	4.2	7.57
Reticulocytes - mm^3^	55.80	41.7	1.34
Total leukocytes - mm^3^	50.80	29.1	1.75
Lymphocytes - mm^3^	58.60	11	5.33
Monocytes - mm^3^	70.70	44.6	1.59
Neutrophils - mm^3^	18.90	49.8	0.38
TNF-α - pg/mL/g	90.60	18	5.03
IL-10 - pg/mL/g	75.70	12.8	5.91
IFN-γ - pg/mL/g	77.60	8.3	9.35
IL-12p40 - pg/mL/g	85.40	13.6	6.28
IL-17 - pg/mL/g	82.90	19	4.36
Cell number - mm^2^	49.80	13	3.83
Number of nests - cm^2^	46.80	76.8	0.61
**Mean ± SD**	59.20 ± 22.80	32.10 ± 25.00	3.72 ± 4.75
**Median**	59.50	24.20	2.24
**Minimum**	8.50	2.50	0.24
**Maximum**	95.00	4.90	26.88
**P-value (Paired samples)**	—	—	<.001
**Effect size (Cohen’s d) - %**	—	—	88.50

BUN, blood urea nitrogen; GOT, glutamate oxaloacetate transaminase; SGPT, glutamate pyruvate transaminase; ALP, alkaline phosphatase; CPK, creatine phosphokinase; CKMB, creatine kinase myocardial band; A/G ratio, albumin to globulin ratio; Crcl, creatinine clearance; MCV, mean corpuscular volume; MCH, mean corpuscular hemoglobin; TNF, tumor necrosis factor; IL, interleukin; IFN, interferon; SD, standard deviation. Significance level = 5% (paired samples t-test).

In paired analysis, significant differences were observed between the effect sizes of the two strains (*p* <.001), with a Cohen’s *d* of 88.50%. The mean of effect sizes for the wild-type subgroup was 59.20 (SD = ± 22.80), whereas for the knockout group it was 32.10 (SD = ± 25.00), with a difference of 54.22% between the means. For 29 of the 36 parameters evaluated (80.55%), the relationship between the effect sizes in the wild-type and knockout groups was greater than 1 (**Table 4**).

Conversely, the effect sizes for plasma CPK (1.03×), plasma albumin (1.29×), plasma globulin (1.21×), the A/G ratio (1.04), urinary creatinine (4.25×), neutrophil count (2.63×), and cardiac tissue cell number (1.64×) were higher in the immunosuppressed subgroup (**Table 4**).

## Discussion

Chagas disease is a diversified form of anthropozoonosis in terms of its ability to generate lesions owing to variability in pathophysiological processes. Attempts have been made to elucidate different mechanisms underlying the pathogenesis of Chagas disease to develop more assertive interventions or efficient prophylactic measures. In the present study, we reported the impact of *T. cruzi* inoculum concentration on the effect sizes of clinical laboratory parameters in acute infection in mice with immunosuppression due to iNOS pathway inactivation.

The imbalance of laboratory markers or the intensity of inflammation in acute *T. cruzi* infection was observed to be inoculum-dependent: the greater the inoculum load, the more severe the changes observed. High inoculum concentrations have previously been reported to correspond to a greater degree of changes in immunocompetent mice, although the responsiveness was reported to be greater at intermediate (medium) or low concentrations in some studies (20–22). Some parameters in immunocompetent animals garnered attention because their effect sizes were affected by more than 80% by the inoculum; some examples are the decreases in the A/G ratio (91.20%) or hematological parameters such as the number of erythrocytes (95%), hemoglobin concentration (91%), hematocrit percentage (93.90%), and concentrations of proinflammatory cytokines such as TNF-α (90.60%) and IL-17 (82.90%).

In the present study, two factors were observed to contribute to a reduction in the A/G ratio. The first was the reduction in plasma albumin concentrations, which may have resulted from an increase in the mobilization of amino acid residues for the synthesis of other proteins and an increase in albumin uptake for mediating different functions, primarily transport. In a study conducted on children under 13 years of age in the acute phase of Chagas disease in an endemic area of Bolivia, a significant increase in alpha-2-macroglobulin and C-reactive protein concentrations was observed (40); this mobilization probably directly affected the albumin levels. The elevation in globulin concentration was an additional indirect contributing factor in the relationship observed in our study. Globulin concentration is expected to be elevated in certain types of infections, including *T. cruzi* infection, wherein there is a consistent increase in the γ-globulin fraction (41, 42), which also affects the reduction in the A/G ratio. Furthermore, we believe that the monitoring and regulation of albumin concentration, if necessary, can favor certain metabolic processes in patients with acute Chagas disease, as has been reported in interactions involved in the mobilization of hematopoietic stem cells in mice (43); however, other types of research, including clinical studies, are warranted.

Hematological alterations caused by *T. cruzi* infection were reported in an evaluation of the medical records of 103 patients in Colombia; the authors observed anemia, along with leukocytosis (17.4% of patients), leukopenia (7.7% of patients), and increased GOT (68.9% of patients), SGPT (50.5%), and creatinine (48.5%) levels in 22.3% of patients (23). In the present study, we confirmed the relationship between hematological effects and *T. cruzi* infection and reported the variability of these effects based on *T. cruzi* inoculum concentration. One of the factors potentially affecting the parameters of the red blood cells is the elevation in the levels of cytokines, such as TNF-α. TNF-α has previously been reported to be associated with the onset of anemia owing to its ability to decrease the survival of erythrocytes and affect the medullary bioavailability of iron (44). The onset of anemia is observed in inflammatory bowel disease, wherein TNF-α concentrations also increase; anti-TNF monoclonal antibody therapy reportedly improves the anemic state in this disease (45).

Several systemic and local changes observed in acute *T. cruzi* infection are mediated by the expression and release of pro-inflammatory cytokines. Observed in the initial phase of the infection are typical macrophage activity, driven by IFN-γ elevation and potentiated by TNF-α activity, and elevation in the levels of other cytokines, such as IL-17, in response to parasitemia (46). The intensity of the immune response is influenced by the availability of antigens with high immunogenicity and the host’s responsive capacity and thus varies for different *T. cruzi* strains and host profiles. In addition, the present study demonstrated the discrepancies in the concentrations of proinflammatory cytokines among subgroups infected with different inoculum concentrations. Cytokine elevation is important for reactive oxygen species (ROS) generation and for increasing the bioavailability of cytokines in phagosomes containing the parasite (14). Consequently, it is essential to assess the impact of these alterations in animals immunosuppressed *via* inactivation of the iNOS pathway, which participates in the production of ROS, including NO (47).

NO affects *T. cruzi* by chemically modifying cysteine-containing proteins and/or by binding to metalloproteins that mediate crucial metabolic processes (10). Other mechanisms that contribute to increased free radical production as well as parasitemia control are phagocytic activity and pathogen pattern recognition, which trigger the assembly of the NADPH oxidase 2 complex and induce the formation of flavocytochrome b558. The active site of the NADPH oxidase 2 complex is oriented toward the phagosome lumen and catalyzes high levels of superoxide production at the expense of oxygen and NADPH; this induces moderate direct toxicity against *T. cruzi*. In addition, the pathway contributes to the generation of second derivatives of ROS such as hydrogen peroxide, which, at high concentrations, promote critical oxidative modifications (48–50). Conversely, among the different mechanisms adopted, the parasite’s adaptations for immune system evasion indicate its attempt to regulate the iNOS expression pathway. The growth of *T. cruzi* in macrophages is dependent on several factors, including its ability to upregulate arginase activity, which consequently leads to competition with iNOS for L-arginine, leading to production of L-ornithine and urea and reduction in NO levels (51, 52). However, despite the clear association between increases in ROS levels and control of parasitemia, a relationship has been established between the absence of ROS and decreases in intracellular parasite multiplication in macrophages (5), which generates doubts about the actual role of the effects of ROS in the infection. Another theory suggests that the increase in parasitism may be associated with the induction of the Wnt signaling pathway by *T. cruzi*. On one hand, the activation of the Wnt pathway corresponds to the replication of the parasite, and on the other hand, the inhibition of the pathway restricts replication and weakens the induction of lethality (53).

In the model of acute *T. cruzi* infection induced by iNOS knockout, similar changes were observed in albumin concentrations, globulin concentrations, and the A/G ratio: albumin concentrations and the A/G ratio decreased, while globulin concentrations increased in an inoculum concentration-dependent manner with effect sizes greater than 80%. Unlike the immunocompetent subgroup, the knockout subgroup did not present effect sizes greater than 80% for any other parameter evaluated.

The wild-type subgroup showed peak parasitemia for the highest inoculum concentration on day 8 after infection, whereas a similar concentration was observed in the knockout subgroup on day 6 of infection, and the peak extended to day 10. In addition to the blood parasites, the amastigote nests in the knockout subgroup occupied a larger area (effect size = 76.8%) than those in the wild-type subgroup. These changes influenced the mortality rates in the knockout subgroup.

The role of deficient iNOS-mediated NO production in the susceptibility of experimental models to *T. cruzi* infection is conflicting. In a study evaluating an iNOS^-/-^ model infected with a reticulotropic lineage strain (*T. cruzi* Tulahuen) for 17 days, the authors reported high susceptibility owing to the absence of NO production, with deaths caused even by intraperitoneal inoculum of low concentrations, and severe histopathological changes (12). Conversely, another study reported that iNOS deficiency was not a limiting factor for resistance to *T. cruzi* infection compared with resistance in wild animals (54). In our evaluation, the absence of iNOS pathway activation affected the relationship between the effect sizes of clinical and laboratory parameters relative to that in the immunocompetent model. In the immunosuppressive model established *via* iNOS pathway inactivation, peripheral monocytes were mobilized, but the inflammatory activity induced by cytokines and associated with cell migration to the target tissues was noticeably affected.

The reduction in the levels of parameters associated with NO production *via* iNOS was also associated with greater vulnerability to *T. cruzi* infection. Reportedly, supplementation with L-arginine increased the effectiveness of immune responses to parasitemia; L-arginine is a semi-essential amino acid necessary for cell proliferation and is the substrate of arginase 1 and iNOS (55).

The major limitations of this study are associated with the molecular complexity of *T. cruzi* infection and the development of Chagas disease. The modification of the evaluated lineage or host immune profile can be linked to other effect sizes with the same parameters described here. Likewise, the effect sizes reported in this study will have limited reproducibility in human models of Chagas disease. However, modeling strategies that take into account the discrepancies in clinical and laboratory parameters can be optimized for and applied to future standardizations, and the models can also be used as indicators of the parasite–host relationship as well as disease prognosis. Therefore, the present study not only contributes to the understanding of the physiopathogenic mechanisms underlying *T. cruzi* infection but also indicate the influence of the concentration of *T. cruzi* during infection and the immunosuppression through the iNOS pathway in clinical laboratory heterogeneity reported in acute Chagas disease.

## Data Availability Statement

The original contributions presented in the study are included in the article/supplementary material. Further inquiries can be directed to the corresponding author.

## Ethics Statement

The protocols for all experiments involving mice were evaluated and approved by the UFTM Institutional Animal Care and Use Committee (protocol number 293/2013).

## Author Contributions

JL-C designed the experiments. WR, CM, LM, TC, and MA performed the experiments. WR, CM, CO, and JL-C analyzed the data. WR, CM, LM, TC, MA, CO, and JL-C wrote the manuscript. All authors contributed to the article and approved the submitted version.

## Funding

CM received doctoral fellowships from CAPES. WR received postdoctoral fellowships from the National Postdoctoral Program of CAPES (Social Demand/PNPD/CAPES). The funders had no role in the study design, data collection, and analysis, decision to publish, or preparation of the manuscript.

## Conflict of Interest

The authors declare that the research was conducted in the absence of any commercial or financial relationships that could be construed as a potential conflict of interest.

## Publisher’s Note

All claims expressed in this article are solely those of the authors and do not necessarily represent those of their affiliated organizations, or those of the publisher, the editors and the reviewers. Any product that may be evaluated in this article, or claim that may be made by its manufacturer, is not guaranteed or endorsed by the publisher.

## Abbreviations

iNOS, inducible nitric oxide synthetase; NO, nitric oxide; IFN-γ, interferon-gamma; ROS, reactive oxygen species; BUN, blood urea nitrogen; GOT, glutamic-oxaloacetic transaminase; SGPT, glutamic-pyruvic transaminase; ALP, alkaline phosphatase; CPK, creatine phosphokinase; A/G ratio, albumin-to-globulin ratio; AIC, Akaike information criterion; BIC, Schwarz Bayesian criterion; OR, odds ratio; CI, confidence interval.
